# Biorefinery of the macroalgae *Ulva lactuca*: extraction of proteins and carbohydrates by mild disintegration

**DOI:** 10.1007/s10811-017-1319-8

**Published:** 2017-10-28

**Authors:** P. R. Postma, O. Cerezo-Chinarro, R. J. Akkerman, G. Olivieri, R. H. Wijffels, W. A. Brandenburg, M. H. M. Eppink

**Affiliations:** 1 Bioprocess Engineering, AlgaePARC, Wageningen University, PO Box 16, 6700 AA Wageningen, the Netherlands; 2 Dipartimento di Ingegneria Chimica, dei Materiali e della Produzione Industriale, Università degli Studi di Napoli Federico II, Piazzale V. Tecchio 80, 80125 Naples, Italy; 3 Nord University, Faculty of Biosciences and Aquaculture, N-8049 Bodø, Norway; 4 Agrosystems Research, Plant Research International, Wageningen University and Research Centre, PO Box 16, 6700 AA Wageningen, the Netherlands

**Keywords:** Macroalgae, Enzymes, Pulsed electric field, High shear homogenization, Osmotic shock, Protein

## Abstract

The effect of osmotic shock, enzymatic incubation, pulsed electric field, and high shear homogenization on the release of water-soluble proteins and carbohydrates from the green alga *Ulva lactuca* was investigated in this screening study. For osmotic shock, both temperature and incubation time had a significant influence on the release with an optimum at 30 °C for 24 h of incubation. For enzymatic incubation, pectinase demonstrated being the most promising enzyme for both protein and carbohydrate release. Pulsed electric field treatment was most optimal at an electric field strength of 7.5 kV cm^−1^ with 0.05 ms pulses and a specific energy input relative to the released protein as low as 6.6 kWh kg_prot_^−1^. Regarding literature, this study reported the highest protein (~ 39%) and carbohydrate (~ 51%) yields of the four technologies using high shear homogenization. Additionally, an energy reduction up to 86% was achieved by applying a novel two-phase (macrostructure size reduction and cell disintegration) technique.

**Electronic supplementary material:**

The online version of this article (10.1007/s10811-017-1319-8) contains supplementary material, which is available to authorized users.

## Introduction

An increasing world population with an estimated 9.6 billion residents by 2050 (United Nations, Department of Economic and Social Affairs, Population Division 2015) is demanding a growth in protein sources in order to feed this number of individuals. The available arable land is becoming limited and will even decrease per capita worldwide (FAO 2011). The sustainable production of food and feed at sea could be an option since the oceans span more than 70% of the planet’s surface (van den Burg et al. 2012) and could thereby address one of the primary issues of securing food production on the Earth (Godfray et al. 2010).

Macroalgae are a potential renewable source of proteins, carbohydrates, chemical building blocks, nutraceuticals, and bioenergy (Holdt and Kraan 2011; van den Burg et al. 2012; van der Wal et al. 2013; van Hal et al. 2014; Wells et al. 2017). They can be divided into three major types including red (Rhodophyta), brown (Phaeophyta), and green (Chlorophyta) algae. Each type has a typical composition which can considerably vary depending on the moment of harvest that results from seasonal variation (Holdt and Kraan 2011). From the three types, the red and green algae contain the highest protein contents (Harnedy and FitzGerald 2011) as well as the highest areal yields (20 t DM ha^−1^) for green macroalgae (van den Burg et al. 2012); therefore, the green macroalgae *Ulva lactuca* was selected as the model species in this work.

*Ulva lactuca*, also known as “sea lettuce”, has a simple morphology and consists of a bilayered cell structure, and the thallus has a flat bladelike appearance (Tan et al. 1999). It is a globally common green seaweed and is able to grow both with a holdfast, e.g., on a rock or free floating.

Algal products are stored inside the cell cytoplasm or are bound to cell membranes and require disintegration before extraction. In addition to the rigid cell wall, macroalgae possess an additional barrier for obtaining the intracellular products, specifically, the macrostructure. On the one hand, this macrostructure aids in the ease of harvesting following cultivation by decreasing the costs of de-watering. On the other hand, it could be imagined that this macrostructure hinders the use of continuous liquid flow processes to disintegrate the cells. Therefore, focus should be placed more towards batch or semi-batch systems in which both the macrostructure and the cell wall are disintegrated. In addition, similar to microalgae, all products should be valorized in a biorefinery approach to make the biomass production economically feasible (Wijffels et al. 2010).

Upon disintegration of the biomass, the mildness of the applied conditions should be taken into account (Vanthoor-Koopmans et al. 2013) in order to prevent negative influences on the product quality. Especially, proteins are sensitive to detrimental conditions such as extreme shear forces, elevated temperatures (i.e., extended heating above 35 °C), or chemicals which cause loss of functional properties. Yet, the complex structure composed of often charged polysaccharides complicates the release of intracellular products (Joubert and Fleurence 2008).

During the past decades, many techniques have been applied to macroalgae biomass for extracting carbohydrates (e.g., ulvans), amino acids, peptides and proteins, pigments, and DNA (Fleurence et al. 1995; Barbarino and Lourenço 2005; Joubert and Fleurence 2008; Sun et al. 2009; Harnedy and FitzGerald 2011, 2013; Alves et al. 2013; Jung et al. 2013; Coste et al. 2015; Polikovsky et al. 2016). To extract those components, mechanical grinding, acid and alkaline treatment, polysaccharidase treatment, high shear forces, osmotic shock, ultrasound, and pulsed electric field (PEF) have been applied. In many of these investigations, mild dried, frozen, or freeze-dried biomass was utilized which is effective for maintaining a consistent supply of biomass. Nonetheless, the biomass may have plausibly already been partially disclosed or permeabilized and consequently have had a detrimental effect of the applied disintegration and extraction method. Therefore, no quantitative data is available from literature on extraction yields of protein or carbohydrates from fresh macroalgae. Moreover, pre-drying or freeze drying the biomass is an energy-intensive process considering a biomass consists of ~ 80% water. With the water heat of evaporation being 0.63 kWh kg^−1^, approximately 2.5 kWh kg_DW_^−1^ (4 kg_water_/kg_biomass_ × 0.63 kWh kg^−1^) is required to remove the water which is equal to about 60% of the biomass energy density (Bruhn et al. 2011). Therefore, the disruption and extraction of proteins and carbohydrates from freshly harvested *U. lactuca* was studied.

The objective of this initial screening study was to provide a strategy to disintegrate freshly harvested *U. lactuca* for the release of water-soluble proteins and carbohydrates using several (non)mechanical methods including osmotic shock, high shear homogenization, enzymatic treatment, and pulsed electric field.

## Materials and methods

### Macroalgae identification, cultivation, and harvest

We identified our *Ulva* samples (origin: Wierderij, Oosterschelde, the Netherlands N 51°41′34.8″, E 3°48′27.5″) according to Stegenga and Mol (1983) and Stegenga et al. (2007) as *Ulva lactuca* L.

The *U. lactuca* was cultivated in 1 m^3^ tanks filled with filtered seawater (Oosterschelde, the Netherlands) at the greenhouse facilities of Wageningen University and Research Centre (Nergena, Wageningen, the Netherlands). The seawater was replaced on a monthly basis. Algae were cultivated and harvested over the period from September 2014 till May 2016. All of the experiments were performed on algae from the same mother culture. For Ultra Turrax experiments, two batches of algae were used and both were harvested in the same period of the following year. For osmotic shock and enzyme experiments as well as for PEF experiments, a single batch of biomass was utilized. The biomass composition of these batches is shown in Table 1.

**Table 1:** Overview of biomass composition. Biomass for Ultra Turrax experiments was harvested around September 2014–December 2015 for osmotic shock, enzyme incubation, and PEF. The biomass was harvested around March–May 2016

Component	Ultra Turrax experiments (*n* = 6, ± SD)	Osmotic shock, enzyme, and PEF experiments (*n* = 3, ± SD)
Moisture (%)	91.4 ± 0.03	81.7 ± 0.012
Protein (% dw)	12.3 ± 1.01	19.8 ± 0.01
Carbohydrate (% dw)	45.8 ± 0.74	45.7 ± 0.60
Ash (% dw)	21.9 ± 0.02	18.7 ± 0.02

After harvest, the biomass was washed using tap water to remove extracellular salts, and extracellular water was drained using a salad spinner. Subsequently, the fresh biomass was directly used or stored no longer than 3 days in sealed bags at 4 °C in the dark.

For each extraction procedure, 5, 10, or 15 g_DW_ biomass per L was prepared in the corresponding medium (see the following sections for details) based on the fresh algae moisture content.

### De-watering

After harvest, the biomass was mildly dried in an oven at 35 °C for 48 h to de-water the algae and then sealed and stored in the dark at room temperature prior to further use.

### Osmotic shock

Osmotic shock experiments to disintegrate the *U. lactuca* were conducted as described by Fleurence et al. (1995) and Harnedy and Fitzgerald (2013) with a number of modifications. Washed *U. lactuca* thallus was suspended in de-ionized water at a biomass concentration of 10 g_DW_ kg^−1^ in a total mass of 5 g (liquid and algae) and stirred gently for up to 24 h (during an overnight period with no stirring) at 4, 22 (RT), or 30 °C. Samples were taken after 1, 4, and 24 h after initiating the osmotic shock. Experiments were performed in duplicate.

### Enzyme treatment

Three commercial available enzymes including Cellulase Onozuka RS (C0615), pectinase Macerozyme R10 (P2401), and β-glucuronidase (SRE0022) were purchased from Sigma-Aldrich (USA), and a freeze-dried powder from abalone gut was kindly provided by Aroma NZ Ltd. (New Zealand). Washed *U. lactuca* thallus was suspended in acetate buffer (pH 4 82 mM HAc, 18 mM NaAc; pH 5 29.5 mM HAc, 70.5 mM NaAc) at a biomass concentration of 10 g_DW_ kg^−1^ in a total mass of 5 g (liquid, algae, and enzyme). Enzyme incubation experiments were conducted for 4 h (preliminary experiments showed no substantial release of proteins or carbohydrates beyond the 4-hour incubation time (the data not shown) according to Table 2 in a shaking water bath. The pH and temperature settings were selected based on the manufacturer optimum conditions. As a control experiment, the same conditions were applied but the enzyme was omitted from the mixture. After incubation, the biomass was removed by centrifugation (20,000*×g*, 10 min), and the supernatant was stored at − 20 °C until further analysis. Experiments were performed in duplicate.

**Table 2:** Overview of enzyme incubation experiments

Exp.	Enzyme	Abbreviation	pH	Temperature (°C)	Concentration (%_DW_)		
a	Cellulase Onozuka RS	CO-RS	5	30	0	0.5	2
b	Pectinase Macerozyme R10	PMC-R10	4	25	0	0.5	2
c	Cellulase Onozuka RS + Pectinase Macerozyme R10	CO-RS + PMC-R10	5	30	0	0.25 (each enzyme)	1 (each enzyme)
d	β-Glucuronidase	β-G	4	30	0	0.5	2
e	Abalone powder	*Ab*	5*	30	0	0.5	2

*Preliminary experiments showed an optimal pH of 5 for abalone powder (data not shown)

### Pulsed electric field

A batch electroporator (Gene Pulser Xcell, Bio-Rad, USA) with cuvettes (gap distance 4 mm, PulseStar, the Netherlands) was used for the electroporation of the *U. lactuca* thallus. The effect of the electric field strength (*E*) was assessed by altering the voltage between 1.2 and 3.0 kV. Additionally, the treatment time was varied by changing the pulse duration (0.05, 0.5 or 5 ms) of the square-wave pulses for a fixed pulse number (two pulses). The *U. lactuca* thallus was suspended at a fixed biomass concentration of 10 g_DW_ kg^−1^ in a 0.04% NaCl solution to obtain a substance with a conductivity of 1250 μS cm^−1^. After electroporation, product release was measured after 1 hour of resting time which allowed the intracellular products to diffuse in the aqueous bulk. These experiments were performed in duplicate.

The specific energy consumption per unit of volume *W*_*V*_ (Frey et al. 2013) and the specific energy consumption per unit of mass *W*_*M*_ are calculated according to:

1 $$ {W}_V\ \left( kWh\ {m}^{-3}\right)=\frac{E^2\bullet {t}_p\bullet N\bullet \sigma }{3600000} $$

2 $$ {W}_M\left( kWh\ {kg}^{-1}\right)=\frac{W_V}{C_x} $$

in which *E* is the electric field strength (V m^−1^), *t*_*p*_ is the pulse length (s), *N* is the number of pulses, *σ* is the electrical conductivity (S m^−1^), and *C*_*x*_ is the biomass concentration (kg m^−3^).

### High shear homogenization

High shear homogenization (HSH) was performed using an Ultra Turrax (T-50, IKA Works, Germany) equipped with a G65F rotor-static dispersing element. Two independent quantitative parameters (biomass concentration *C*_*x*_ and rotor tip speed *u*_*s*_) were studied at three levels in a design of experiments similar to Postma et al. (2015). Modde v.9.1 (Umetrics, Sweden) DOE software was utilized to study the effect on *C*_*x*_ and *u*_*s*_ using a central composite face-centered design (CCF). In this design, the experimental variation of the triplicate center experiment is extrapolated to the low and high values. The experimental range and parameters are depicted in Table 3.

**Table 3:** Parameters of experiments for CCF design of HSH experiment

Parameters	Factor	Low value (−1)	Center value (0)	High value (+1)
Biomass concentration (g kg^−1^)	×1	~ 5.4	~ 10.7	~ 16.1
Rotor tip speed (m s^−1^)	×2	11	16	21

A third (multilevel) parameter (X3), i.e., pre-treatment by manual cutting, was also evaluated to assess the ability of the HSH to address the macrostructure of the *U. lactuca* thallus. The thalli were either left intact (F, X3 240 cm^2^), cut into pieces of ~ 3 × 4 cm (FC, X3 12 cm^2^), or cut into pieces of 1 cm^2^ (FC2, X3 1 cm^2^). For each of the pre-cut conditions, the above mentioned CCF was conducted. For all of the experiments, the macroalgal biomass was suspended in phosphate buffered saline (PBS) (1.54 mM KH_2_PO_4_, 2.71 mM Na_2_HPO_4_∙2H_2_O, and 155.2 mM NaCl at pH 7.0) to a total volume of 0.25 L.

The temperature during the treatment was controlled by placing the beaker glass in a water-ice suspension whereby the temperature never exceeded 35 °C for an experiment of a maximum of 40 min.

Energy consumption was measured by means of an energy logger (Energy Logger 4000, Voltcraft, Germany). The specific energy consumption *E*_*M*_ is defined as the consumed energy per kg of dry biomass (kWh kg_DW_^−1^).

### Analytical methods

#### Biomass dry weight content

Biomass dry weight determination was conducted by taking a known amount of washed fresh weight *U. lactuca* (~ 2 g) and placing it in pre-weighted aluminum cups. The cups were incubated overnight at 105 °C and re-weighted. Dry weight determination was performed in technical triplicate.

#### Protein analysis

Water-soluble protein release and total protein content on biomass DW was determined according to Postma et al. (2015). For total protein content on DW, 6 mg of freeze-dried algae were bead beaten in 1.0 mL lysis buffer I (60 mM Tris, 2% SDS, pH 9.0) in a lysing matrix D tube (6913–5000, MP Biomedicals Europe, France). The tubes were beaten using a bead beater (Precellys 24, Bertin Technologies, France) for three cycles of 60 s at 6500 rpm with 120 s breaks between cycles. The water-soluble protein content was analyzed by obtaining supernatant from treated samples and was diluted twice using a lysis buffer II (120 mM Tris, 4% SDS, pH 9.0). Subsequently, samples for both the total protein content and water-soluble protein content were incubated at 100 °C for 30 min before quantification using a commercial kit (DC Protein assay, Bio-Rad, USA). Bovine serum albumin (A7030, Sigma-Aldrich, USA) was utilized as the protein standard, and the absorbance was measured at 750 nm. The protein yield (*Y*_*p*_) was expressed as:

3 $$ {Y}_p=\frac{C_{p,\sup }}{C_{p,\mathrm{biomass}}} $$

where *C*_*p,*sup_ is the protein content in the supernatant (%_DW_) and *C*_*p*,biomass_ is the total protein content on DW (%_DW_).

For soluble proteins, protein values found in control experiments with an enzyme but without algae were subtracted from the water-soluble protein release samples. Protein analysis was performed in technical duplicate.

#### Carbohydrate analysis

The water-soluble carbohydrate and total carbohydrate on DW analyses were conducted as previously described (Postma et al. 2016). For total carbohydrate analyses, 1 mg of DW biomass was hydrolyzed in 1 mL 2.5 M HCl in a heating block at 100 °C for 3 h. Samples were neutralized with 1 mL 2.5 M NaOH. Samples for total carbohydrates and soluble carbohydrates in the supernatant were analyzed according to DuBois et al. (1956), and 0.2 mL of 5% *w*/*w* phenol and 1 mL of concentrated sulfuric acid were added to 0.2 mL of the sample. The samples were incubated at 35 °C for 30 min before reading the absorbance at 485 nm against a blank of 0.2 mL 5% *w*/*w* phenol, 1 mL concentrated sulfuric acid, and 0.2 mL of de-ionized water. Glucose was used as a standard. The carbohydrate yield (*Y*_*c*_) was expressed as:

4 $$ {Y}_c=\frac{C_{c,\sup }}{C_{c,\mathrm{biomass}}} $$

in which *C*_*c*,sup_ is the carbohydrate content in the supernatant (%_DW_) and *C*_*c*,biomass_ is the total carbohydrate content on DW (%_DW_). A carbohydrate analysis was performed in technical duplicate.

### Scanning electron microscopy

Macroalgae thallus treated by HSH was fixed on poly-L-lysine-coated cover slips (Ø 8 mm) by applying a drop of 150 μL on the cover slip and incubating for 1 h. Thereafter, the glasses with attached cells and thalli treated by enzymes were rinsed by dipping them in fresh PBS and subsequently fixed for 1 h in 3% glutaraldehyde in PBS. After being washed twice in PBS, the samples were postfixed in 1% OsO_4_ for 1 h, rinsed with demi water, and dehydrated in a graded (30–50–70–90–100–100%) ethanol series. Subsequently, cover slips and thalli were critical-point dried with carbon dioxide (EM CPD 300, Leica, Wetzlar, Germany). The cover slips with cells and the thalli were fit on sample holders using carbon adhesive tabs (EMS, Washington, USA) and sputter coated with 10 nm Wolfram (EM SCD 500, Leica, Germany). The cells and thalli were analyzed at room temperature in a high-resolution scanning electron microscope at 2 KV (Magellan 400, FEI, the Netherlands). Images were contrast enhanced with Photoshop CS5.

### Statistical analysis

Statistical analysis was performed with Modde DOE software (Umetrics, Sweden) or by analysis of variance (ANOVA) in Excel (Microsoft, USA). Significant differences within groups were determined with independent sample *t* tests at a significance level of 95%.

## Results and discussion

The protein and carbohydrate yields presented in this work are based on the measured total protein and carbohydrate composition of the *U. lactuca* biomass (Table 1). Due to seasonal variation, the protein content for the first set of experiments (Ultra Turrax) was observed to be significantly lower compared to the second set of experiments (osmotic shock, enzyme incubation, and PEF). First, the results of extraction utilizing osmotic shock are presented followed by the effects of enzymatic disintegration, PEF treatment, and high shear homogenization.

### De-watering

Preliminary experiments using mild (35 °C, 48 h) pre-dried biomass prior to disintegration by HSH showed a lower protein yield (5.6% ± 1.7, *n* = 6) compared to the use of fresh biomass (15.9% ± 6.7, *n* = 6). The ANOVA revealed that this was a significant (*p* = 0.004) difference. Therefore, in subsequent experiments, only fresh biomass was used.

### Osmotic shock

**Fig. 1:**
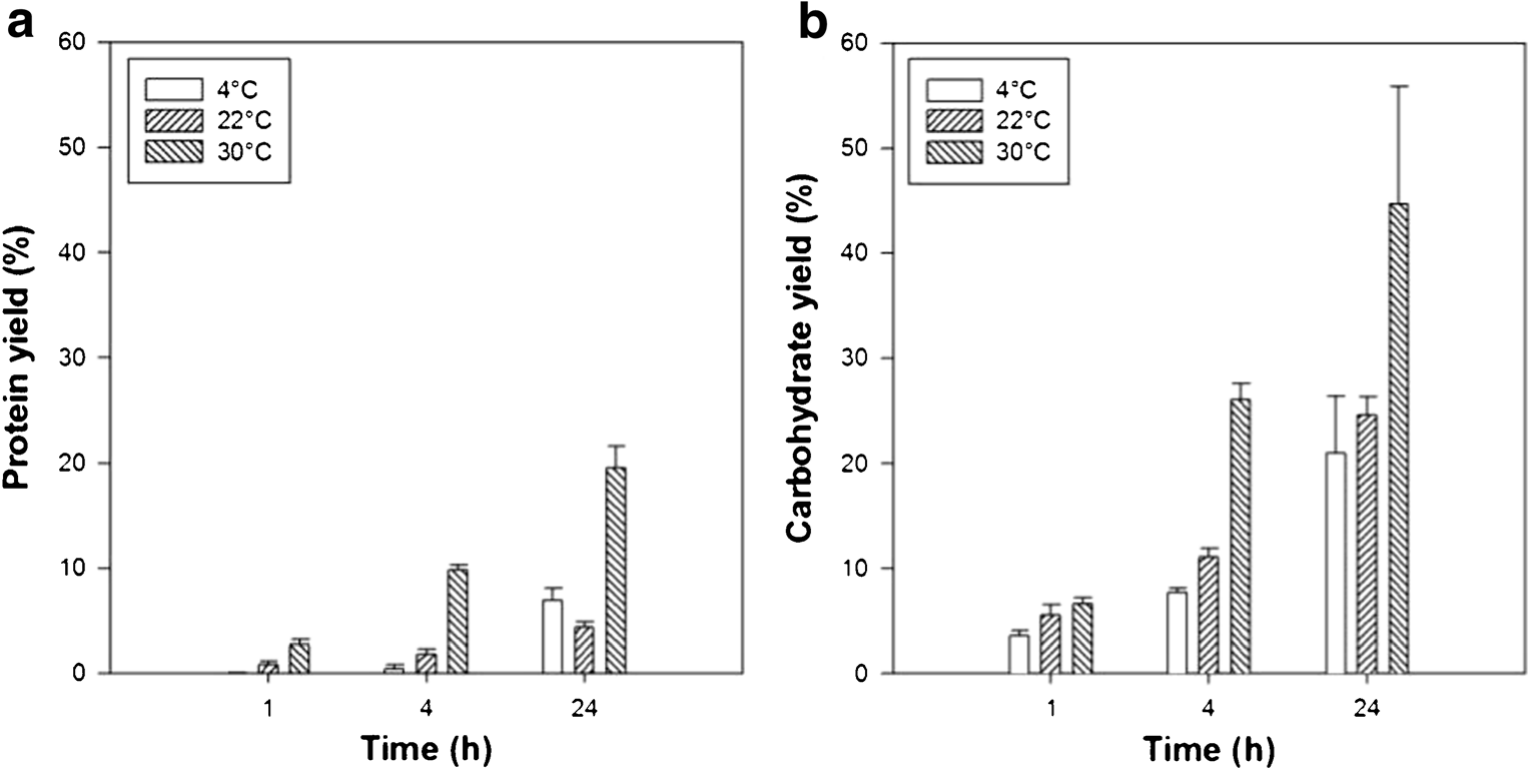
Overview of protein and carbohydrate yields as a function of temperature and duration following osmotic shock. Biomass concentration 10 g_DW_ kg^−1^. Error bars show the standard deviation, *n* = 2

The results of the osmotic shock on the release of proteins and carbohydrates are shown in Fig. 1. Both the temperature and the duration had a substantial effect on the release of soluble proteins and carbohydrates. Using the independent sample *t* test, it was determined that increasing the time of the osmotic shock from 4 to 24 h significantly increased the product release at 4 °C (*p =* 0.017), 22 °C (*p =* 0.037), and 30 °C (*p =* 0.024). An increase of the temperature from 4 to 22 °C did not result in any notable improvement of the product release independent of the time of incubation (*p > 0.05*); however, further increasing the temperature to 30 °C (from either 4 or 22 °C) did (*0.002 < p < 0.042*). The greatest protein (19.5%) and carbohydrate (44.7%) yields were observed when the temperature was increased up to 30 °C in combination with an incubation time of 24 h. Harnedy and Fitzgerald (2013) found only minimal effect of both the temperature (4 or 22 °C) and the duration (3, 7, or 16 h) on the aqueous extraction of protein from *Palmaria palmata* following the osmotic shock. A maximum yield of 5.9% was ascertained at a temperature of 4 °C after 7 h of incubation while, in this work, a similar yield was only achieved after 24 h. Yet, at a temperature of 22 °C and a duration of 24 h, the yield of 4.3% in this work is similar to the yield of 4.7% found by Harnedy and Fitzgerald (2013) at 22 °C after 16 h.

### Enzymatic degradation of cell wall

**Fig. 2:**
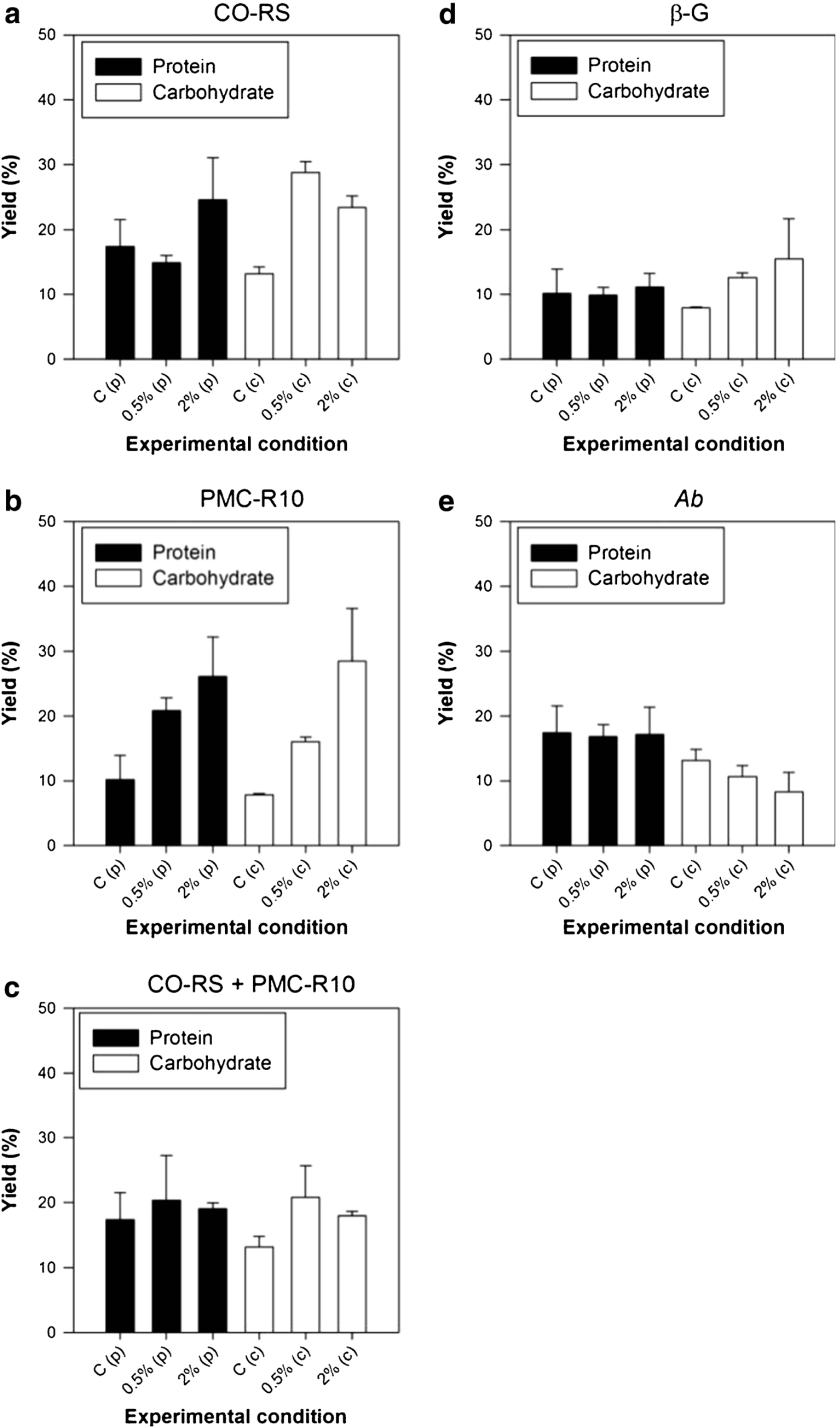
Protein and carbohydrate yields for Control “C”, “0.5%_DW_”, and “2%_DW_” enzyme dosage using Cellulase Onozuka RS (**a**), Pectinase Macerozyme R-10 (**b**), Cellulase + Pectinase (**c**), β-glucuronidase (**d**), and abalone powder (**e**). Error bars show the standard deviation, *n* = 2

Enzymatic incubation was assessed as a mild approach with respect to HSH because of its high presumed energy consumption. Four different enzymes and one mixture were screened; the results of the protein and carbohydrate yield are shown in Fig. 2. The control experiments in which no enzyme was added to the fresh *U. lactuca* thallus demonstrated a relatively high yield with the enzyme-incubated experiments. The major contributor to this was the low osmolarity of the applied acetate buffer (20 mOsm) while seawater has a typical osmolarity of 1000–1200 mOsm. This low osmolarity most likely caused an osmotic shock which caused a part of the cells to break.

The applied CO-RS and PMC-R10 show the highest yields (~ 25–30%) for both protein and carbohydrates at a crude enzyme concentration of 2% (Fig. 2a, b). Increasing the crude enzyme concentration from 0.5 to 2% did not result in a proportional increase of the protein or carbohydrate yield (i.e., a fourfold increase in enzyme did not result in a fourfold yield increase). It should be mentioned that PMC-R10 also contains cellulose and hemicellulose activities (according to manufacturer information) which might have a slight effect on the experimental outcome. It is worth mentioning, the results of Reddy and Fujita (1991) who found that the addition of 2% PMC-R10 did not result in protoplast release. Moreover, it was mentioned that no effect on the cell wall was observed at all. Remarkably, the mixture of both enzymes (CO-RS and PMC-R10) as a cocktail (Fig. 2c) did not result in an improvement of the yield. This is in accordance with the work of Reddy et al. (2006) who applied 2% CO-RS and a mixture of 2% CO-RS + 2% PMC-R10 to several *Ulva* sp*.* and found that the generation of protoplasts was more effective without pectinases. Enzymatic incubation using β-glucuronidase (β-G) resulted in a protein yield (~ 10%) and carbohydrate yield (~ 15%) which were both relatively low. Moreover, no improvement with respect to the control was observed. This was unexpected since β-G is an enzyme that is isolated from the entrails of abalone, a natural grazer of *U. lactuca*. *Ab* powder also demonstrated poor protein yields (~ 18%) and carbohydrate yield (~ 9%) at the same level as the control experiment. This abalone powder was received dried; therefore, enzymes were possibly denatured during the extraction of the entrails prior to delivery, or the enzyme specificity was not able to disintegrate the *U. lactuca* macrostructure. In contrast, Reddy and Fujita (1991) found that abalone powder was able to degrade the cell wall of three *Enteromorpha* spp. (taxonomic synonym for *Ulva* spp). but not *U. lactuca*. Fleurence et al. (1995) determined that a mixture of cellulase, hemicellulase, and β-glucanase was effective on *Ulva rigida* and *Ulva rotundata* with protein yields of 18.5 and 22.0%, respectively. On the contrary, they observed protein yields below 1% for a commercial cellulase.

### Pulsed electric field

**Fig. 3:**
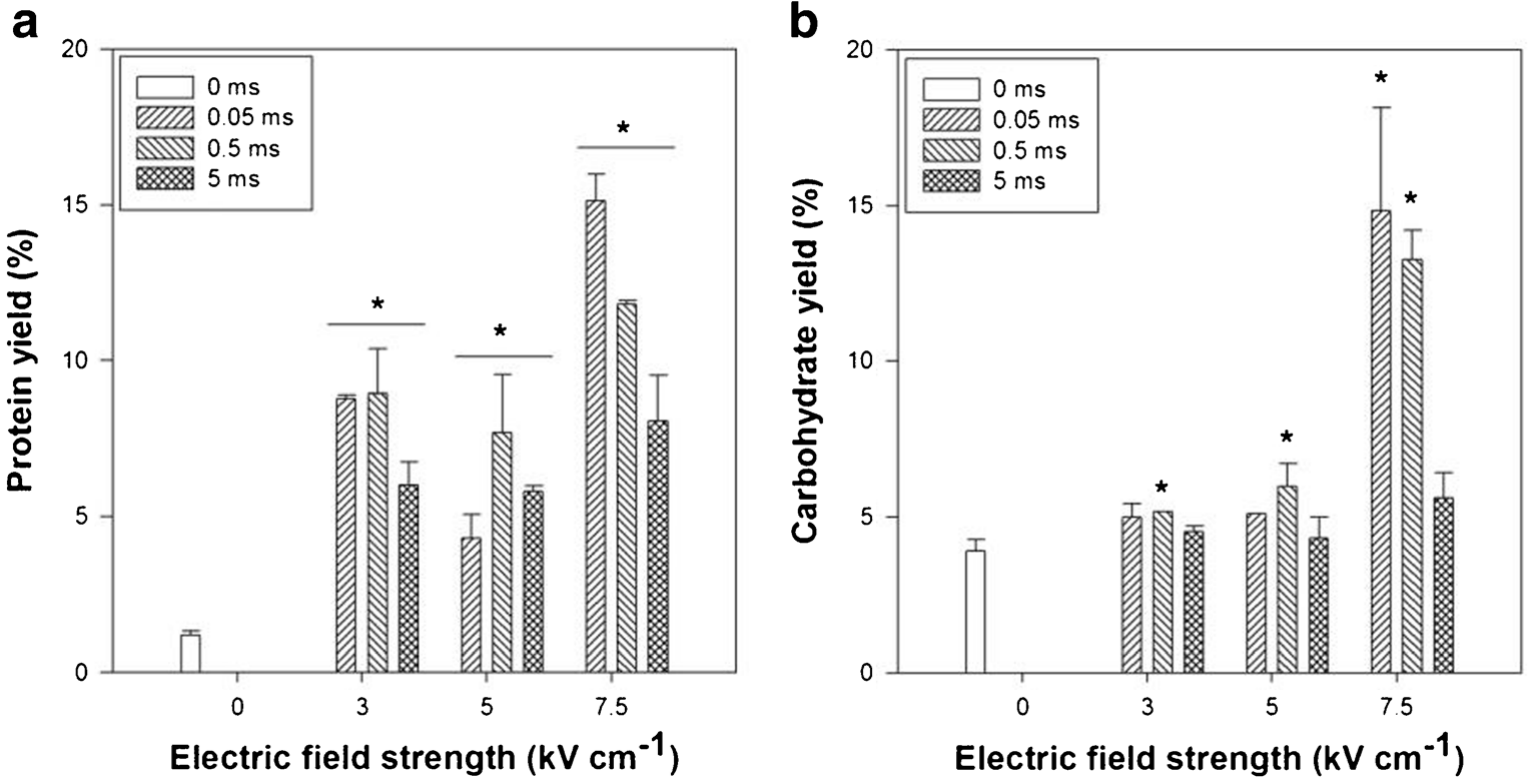
Protein yield (**a**) and carbohydrate yield (**b**) as a function of electric field strength and pulse duration during PEF treatment. * Significant difference from control (0 kV cm^−1^). Error bars show the standard deviation, *n* = 2

The effect of a PEF treatment on the release of water-soluble protein and carbohydrates is illustrated in Fig. 3. Under all of the pulse conditions, an increase in the water-soluble protein (Fig. 3a) content was observed in the supernatant with respect to the control (0 kV cm^−1^). The ANOVA revealed that the effect of the PEF treatment was significant (*p* < 0.05) for each condition. At both 3 and 5 kV cm^−1^, the pulse duration had no significant effect on the protein yield. The highest protein yield of 15.1% was achieved at 7.5 kV cm^−1^ using 0.05 ms pulses. Moreover, at 7.5 kV cm^−1^ and 0.05 ms pulses (pulse number 2), the *t* test showed a significantly higher protein yield compared to 0.5 (*p* = 0.019) or 5 (*p* = 4.95∙10^−4^) ms pulses. When examining only the effect of the electric field strength at a fixed pulse duration of 0.05 ms, at 7.5 kV cm^−1^, a higher protein yield was obtained compared to 3 (*p* = 0.015) or 5 (*p* = 0.004).

Polikovsky et al. (2016) applied an electric field strength of 2.964 kV cm^−1^ and found a specific energy input relative to the extracted protein of 251 ± 3 kWh kg_PROT_^−1^. Taking into account the highest protein yield in this study (15.1%) with the corresponding specific energy input of 0.2 kWh kg_DW_^−1^, a specific energy input relative to the extracted protein of only 6.6 ± 0.28 kg_PROT_^−1^ was obtained.

The carbohydrate release (Fig. 3b), on the other hand, showed a less significant effect. With respect to the control, a pulse duration of 0.5 ms in combination with an electric field strength of 3, 5, or 7.5 kV cm-1 resulted in a notable increase of the carbohydrate yield (*p* < 0.05). The highest carbohydrate yield was obtained in the same conditions as the highest protein yield, specifically, 0.05 ms and 7.5 kV cm^−1^.

The spontaneous release of protein and carbohydrates in the control could be caused by the lack of osmotic pressure since the osmolarity of the used PEF medium was only 13 mOsm (0.04% *w*/*w* NaCl solution). Such a low osmolarity was required to ensure a low conductivity of the PEF medium in order to avoid sparking. When examining the osmotic shock data from the “Osmotic shock” Section, the time scale during PEF was much shorter yet the incubation afterwards (at RT) to allow for diffusion was 1 h which resulted in similar yields.

### High shear homogenization

**Fig. 4:**
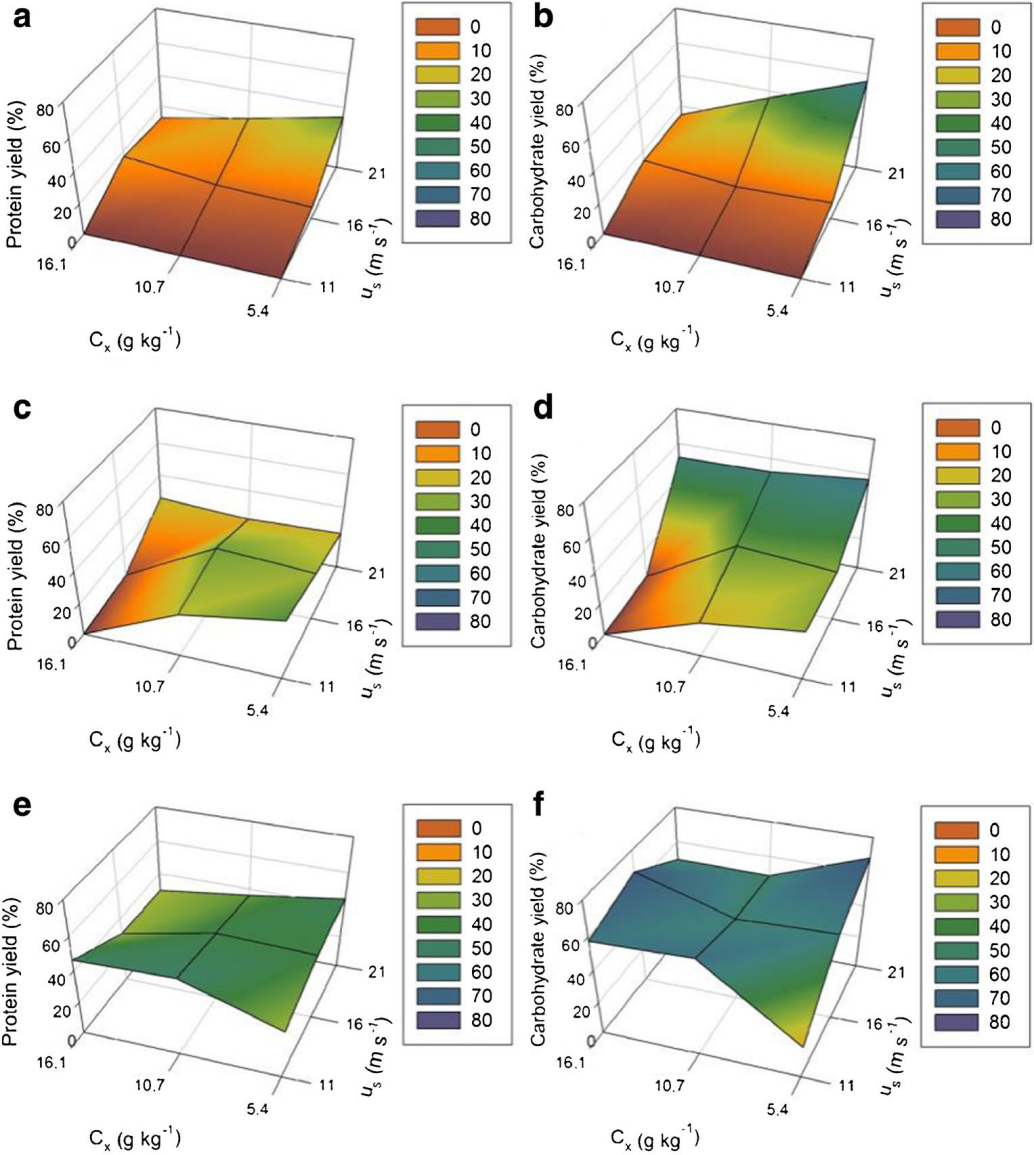
3D mesh plots of protein yield using uncut “F” (**a**), 3 × 4 cm pieces “FC” (**c**), 1 × 1 cm pieces “FC2” (**e**), biomass and carbohydrate yields using uncut “F” (**b**), 3 × 4 cm pieces “FC” (**d**), 1 × 1 cm pieces “FC2” (**f**) biomass. Color coding in the legend represents the protein and carbohydrate yield (%). Protein and carbohydrate content measured in the supernatant after 40 min of disintegration, *n* = 2

To determine the ability of the HSH to disintegrate both the macrostructure and the cellular structure (i.e., cell wall), the fresh biomass was either left intact (“F”) or manually pre-cut to pieces of 3 × 4 (“FC”) or 1 × 1 cm (“FC2”) preceding the HSH treatment. The results of the protein and carbohydrate yields are shown in Fig. 4.

The first observation made for the uncut “F” biomass (Fig. 4a, b) was that the thallus could not be effectively reduced in size under all conditions. The thallus remained intact at especially low or moderate rotor speeds, and thus, also did not result in disintegration of the cell wall to release proteins and carbohydrates (*Y*_*P*_ 0%). Increased rotor speeds up to 21 m s^−1^ in combination with low biomass concentrations resulted in a maximum protein and carbohydrate yield of 34 and 65%, respectively.

When the *U. lactuca* thallus was pre-cut into pieces of 3 × 4 cm “FC” (Fig. 4c, d), it was observed that the thallus was more easily reduced in size except for the combination of high biomass concentration and a low rotor speed. A low to moderate biomass concentration combined with a low to moderate rotor speed resulted in the highest protein yields up to 37% while a high rotor speed was still favorable for the release of carbohydrates up to a 57% yield. This shows that the maximal yield did not improve; however, the thallus and cell wall were more easily disintegrated over a broader range of operating conditions.

For the smallest pieces of 1 × 1 cm “FC2” (Fig. 4e, f), the thallus was disintegrated under all conditions. The highest protein yields were obtained for either moderate to high biomass concentrations combined with a relatively low rotor speed or a low to moderate biomass concentration and relatively high rotor speed. The maximum protein yield improved up to 48% for a biomass concentration of 10.7 g kg^−1^ and a rotor speed of 11 m s^−1^. The carbohydrate yields improved under all conditions except for a high rotor speed and low biomass concentration with a maximal yield of 68% for a low biomass concentration of 5.4 g kg^−1^ and a rotor speed of 21 m s^−1^. Using a similar HSH principle as that utilized by Harnedy and Fitzgerald (2013) who studied the extraction of protein from pre-frozen and dried (50 °C) *Palmaria palmata*, a water-soluble protein yield of 3–4% was achieved which could be improved up to 40% in combination with a sequential alkaline extraction to disintegrate the proteins as well as hydrolyses.

The results from Fig. 4 show that the macrostructure of *U. lactuca* was initially limiting the release of the water-soluble proteins and carbohydrates. When the thallus was manually pre-cut, the release of both proteins and carbohydrates improved. Moreover, using high rotor speeds increased the energy consumption, with 40 min of disintegration at 11, 16, or 21 m s^−1^ with a biomass concentration of 10.7 g kg^−1^ required 30.4, 57.3, or 77.8 kWh kg_DW_^−1^. To reduce energy consumption and eliminate the manual pre-cutting, a two-phase HSH strategy was developed. This two-phase strategy included a cutting (i.e., thallus size reduction) phase of 3.5 min with a high rotor speed (21 m s^−1^) and a disintegration phase to release the proteins and carbohydrates at either 11 or 16 m s^−1^.

**Fig. 5:**
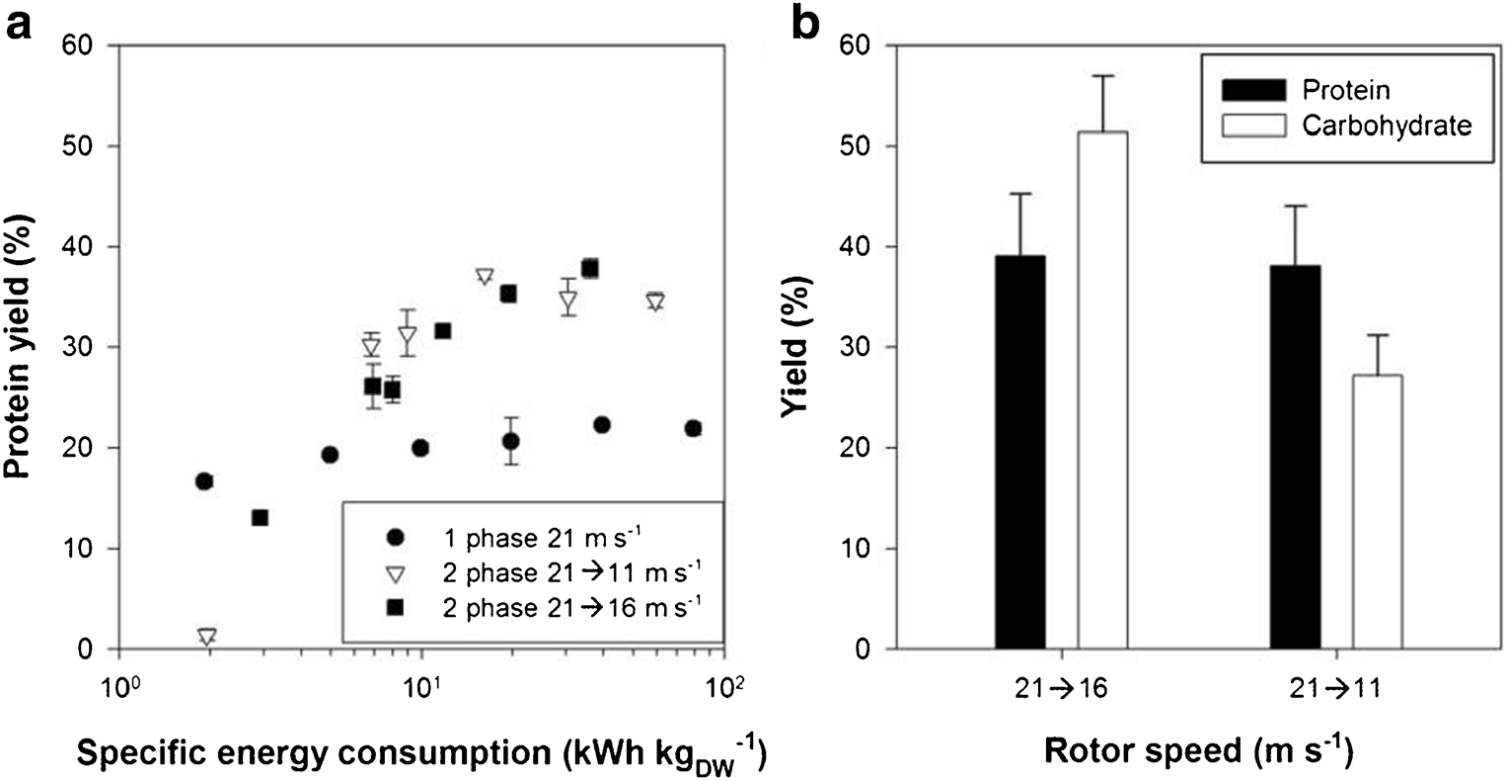
Protein yield (%) as a function of the specific energy consumption (kWh kg_DW_^−1^). Single rotor speed (one phase) of 21 m s^−1^ compared to two-phase (**a**) and maximal protein and carbohydrate yield for the two-phase experiments at 21–16 and 21–11 m s^−1^ (**b**) for a fixed biomass concentration of 10.7 g kg^−1^. Error bars show the standard deviation, *n* = 2

Figure 5a shows the protein yield as a function of the specific energy consumption. Similar to what was observed in the single phase experiments where the thallus was manually pre-cut (Fig. 4c–f), the maximum protein yields were higher in the two-phase experiment than in the single-phase experiment at high speed (Fig. 4a). This substantiated that the initial pre-cutting phase of 3.5 min was sufficient to reduce the size of the thallus and allow milder disintegration of the cell wall to release the water-soluble proteins and carbohydrates. Furthermore, it could be observed that both two-phase set-ups resulted in similar protein yields (*p > 0.87*) at equal energy consumption. This was because the set-up at which a moderate speed disintegration phase was used was faster compared to the low speed. On the other hand, when also investigating the carbohydrate yield (Fig. 5b), the maximum carbohydrate yield did differ significantly (*p < 0.05*) with yields of 51 and 27% for the “21 → 16” and “21 → 11” m s^−1^ set-up, respectively. A possible explanation is that the high shear forces not only released natively soluble carbohydrates from the cytosol but also chopped the polysaccharide structure of the thallus. This effect was also observed as shown in Fig. 4D and 3F whereby the carbohydrate yield was lower at 11 m s^−1^ compared to 16 m s^−1^.

Beyond ±10 min of disintegration during the two-phase experiments (11–16 kWh kgDW^−1^), no further increase in the protein yield was occurring. Compared to a full experiment of 40 min at 21 m s^−1^ (77.8 kWh kg_DW_^−1^) with intact thallus, this corresponds to an energy reduction of 79 to 86%. Taking into account the protein yield after ten minutes of disintegration, a specific energy input relative to the extracted protein of 318 ± 48 and 313 ± 71 kWh kg_PROT_^−1^ for the “21 → 16” and “21 → 11” m s^−1^ set-up was obtained, respectively.

Based on the design of the experiments made in the MODDE software, an indicative model was created to predict the protein and carbohydrate yield; the details of this model are included in the supplementary material (S1).

### Scanning electron microscopy

**Fig. 6:**
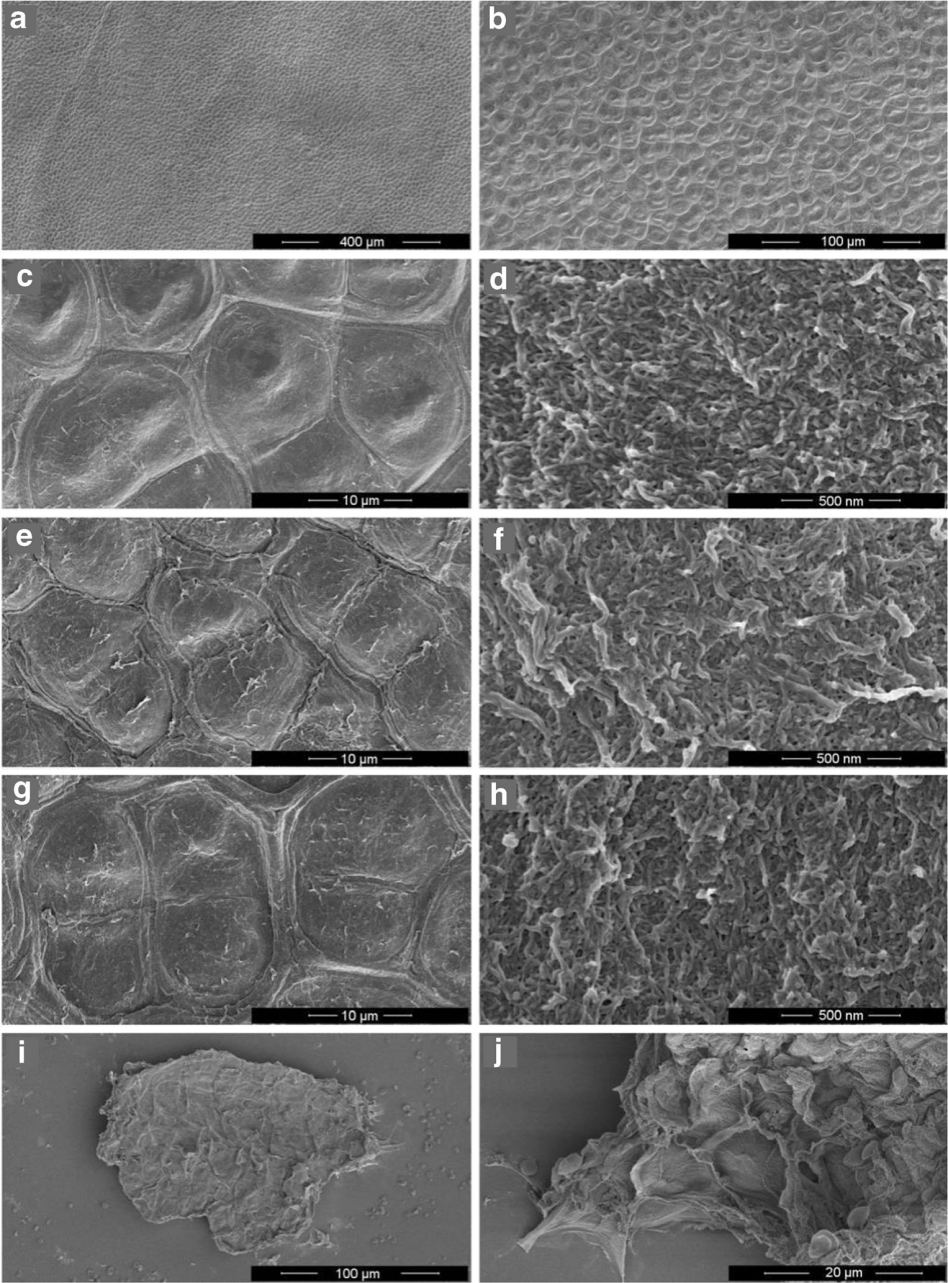
SEM pictures of *U. lactuca* thallus before treatment (**a**, **b**, **c**, **d**), after PMC-R10 treatment (**e**, **f**), after abalone treatment (**g**,**h**), and after HSH (**i**, **j**)

To obtain an improved understanding behind the mechanism of disintegration by enzymatic disintegration and HSH, SEM pictures from *U. lactuca* thallus before and after treatment were taken, untreated thallus after harvest, PMC-R10 and abalone powder enzymatic digestion, and after HSH. Figure 6a–d exhibits the untreated *U. lactuca* thallus. A nice and structured arrangement of the individual cells is visible (Fig. 6a, b) with a clear turgor (Fig. 6c). A detailed picture of the cell wall (Fig. 6d) shows a rough surface.

Treatment of the thallus with the pectinase PMC-R10 revealed a loss of the cell turgor (Fig. 6e); however, no clear destruction of the cells could be observed. Additionally, the cell wall surface (Fig. 6f) does not indicate substantial differences with respect to the control. This accords with the above mentioned findings of Reddy and Fujita (1991) who also did not observe any effect of PMC-R10 on the cell wall surface. Contradictory, the cell wall must be permeable in order to release the proteins and carbohydrates up to a yield of almost 30%. Similar observations were made for the abalone powder treatment with a loss of turgor and no modifications on the cell wall surface (Fig. 6g, h).

HSH of the thallus lead to the highest yields in this work and is clearly demonstrated by the small thallus pieces that were observed (Fig. 6i). Moreover, broken and emptied individual cells could be observed (Fig. 6j).

### Perspective of macroalgae disintegration for biorefinery

Macroalgae show a large potential in terms of securing a world protein supply and also possess interesting carbohydrates. The primary hindrance is being able to retrieve these interesting components due to low digestibility. Only a small number of studies exist on the evaluation of macroalgal disintegration and protein/carbohydrate extraction (Fleurence et al. 1995; Joubert and Fleurence 2008; Harnedy and FitzGerald 2013). This study evaluated four different methodologies in which the release of both proteins and carbohydrates was investigated. A summary of the best results obtained in this work and literature is provided in Table 4.

**Table 4:** Overview of best product yields achieved by osmotic shock, enzyme incubation, PEF, and HSH from current work and literature

Technique	Algal species	Procedure	Protein yield (%) ± STD	Carbohydrate yield (%) ± STD	Reference
Osmotic shock	*U. rigida*	Overnight, 4 °C	9.7 ± 0.6	n/a	(Fleurence et al. 1995)
	*U. rotundata*	Overnight, 4 °C	14.0 ± 1.8	n/a	
	*P. palmata*	7 h, 4 °C	5.9 ± 0.4	n/a	(Harnedy and FitzGerald 2013)
	*U. lactuca*	24 h at 30 °C	19.5 ± 2.1	44.7 ± 11.2	(This work)
Enzyme incubation	*U. rigida*	2 h, 3% cellulase A, 30 °C	18.5 ± 2.1	n/a	(Fleurence et al. 1995)
	*U. rotundata*	2 h, 3% cellulase A, 30 °C	22.0 ± 1.5	n/a	
	*P. palmata*	48∙10^3^ U Shearzyme 500 L + celluclast 1.5 L	18.4 ± 1.7	n/a	(Harnedy and FitzGerald 2013)
	*U. lactuca*	4 h, 2% PMC-R10, 30 °C	26.1 ± 6.0	28.1 ± 8.1	(This work)
PEF	*U. lactuca*	*E* 3 kV cm^−1^, 75 pulses of 5.7 μs	< 1%^a^	n/a	(Polikovsky et al. 2016)
	*U. lactuca*	*E* 7.5 kv cm^−1^, 2 pulses of 0.05 ms	15.1 ± 0.7	14.8 ± 3.3	(This work)
HSH	*P. palmata*	24,000 RPM, 1 h post incubation	4.3 ± 0.1	n/a	(Harnedy and FitzGerald 2013)
	*U. lactuca*	2 phase set-up “21 → 16” m s^−1^	39.0 ± 6.2	51.3 ± 5.6	(This work)

^a^Assumptions 80% moisture content, 16% protein on DW

Osmotic shock would merely require that the biomass is washed with demineralized water and incubated for 24 h at 30 °C. However, such extensive holding times might become an issue when being conducted for large volumes at industrial scales.

Enzymatic disintegration of *U. lactuca* is an effective method for releasing protein and carbohydrates. Yet, it was observed that the applied buffer may possibly cause an osmotic shock depending on the selected conditions. A slight dosage effect was observed (e.g., for PMC-R10); however, no proportional increase was determined when increasing the enzyme concentration from 0.5 to 2% of biomass DW. Therefore, further optimization of the applied buffer system (e.g., osmolarity) and enzyme concentration should be subjects of further study. In addition, further characterization of the abalone gut powder is required in order to obtain a better understanding about the composition and specificity of this natural enzyme mixture.

PEF showed initial promising results though the screening revealed that an electric field strength of 7.5 kV cm^−1^ provided the highest results, which is contradicting the results of Polikovsky et al. (2016) who only required a field strength of ~ 3 kV cm^−1^. This might be due to the difference in the used PEF equipment, and thus, requires further investigation.

This screening study revealed that HSH is the most effective technique among the tested methods to reduce the size of the thallus, disintegrate the cell wall, and result in the highest yields. The applicability of this method on a larger scale should be validated (according to the manufacturer’s website, scaled-up equipment is available). Furthermore, the process revealed to be energy intensive (≥ 11 kWh kgDW^−1^) with respect to the biomass energy density ~ 4.8 kWh kgDW^−1^ (on ash-free dry matter) (Bruhn et al. 2011). The applied biomass concentrations were rather low compared to microalgae disintegration (Doucha and Lívanský 2008; Goettel et al. 2013); therefore, an increase might be the solution to overcome the high specific energy consumption.

To conclude, this research provides a first indication that it is possible to release water-soluble proteins and carbohydrates from fresh *U. lactuca* biomass. In descending order, the highest carbohydrate yields per treatment, HSH (~ 51%) > osmotic shock (~ 45%) > enzyme degradation (~ 28%) > PEF (~ 15%), and, in descending order, the highest protein yields per treatment, HSH (~ 39%) > enzyme degradation (~ 25%) > osmotic shock (~ 20%) > PEF (~ 12%). Nevertheless, PEF (6.6 kWh kg_prot_^−1^) did show a more promising specific energy consumption with respect to the extracted protein compared to HSH (313–318 kWh kg_prot_^−1^). Finally, additional research is required to gain additional understanding about the exact mechanisms behind the screened mild disintegration methods.

## Electronic supplementary material

ESM 1 (DOCX 17 kb)

## References

[CR1] Alves A, Sousa RA, Reis RL (2013). A practical perspective on ulvan extracted from green algae. J Appl Phycol.

[CR2] Barbarino E, Lourenço SO (2005). An evaluation of methods for extraction and quantification of protein from marine macro- and microalgae. J Appl Phycol.

[CR3] Bruhn A, Dahl J, Nielsen HB, Nikolaisen L, Rasmussen MB, Markager S, Olesen B, Arias C, Jensen PD (2011) Bioenergy potential of *Ulva lactuca*: biomass yield, methane production and combustion. Bioresour Technol 102:2595–2604 10.1016/j.biortech.2010.10.010 21044839

[CR4] Coste O, Malta E, López JC, Fernández-Díaz C (2015). Production of sulfated oligosaccharides from the seaweed *Ulva* sp. using a new ulvan-degrading enzymatic bacterial crude extract. Algal Res.

[CR5] Doucha J, Lívanský K (2008). Influence of processing parameters on disintegration of *Chlorella* cells in various types of homogenizers. Appl Microbiol Biotechnol.

[CR6] DuBois M, Gilles KA, Hamilton JK, Rebers PA, Smith F (1956). Colorimetric method for determination of sugars and related substances. Anal Chem.

[CR7] FAO (2011). The state of the world’s land and water resources for food and agriculture (SOLAW)—managing systems at risk. Food and Agriculture Organization of the United Nations.

[CR8] Fleurence J, Coeur CL, Mabeau S, Maurive M, Landrein A (1995) Comparison of different extractive procedures for proteins from the edible seaweeds *Ulva rigida* and *Ulva rotundata*. J Appl Phycol 7:577–582

[CR9] Frey W, Gusbeth C, Schwartz T (2013). Inactivation of *Pseudomonas putida* by pulsed electric field treatment: a study on the correlation of treatment parameters and inactivation efficiency in the short-pulse range. J Membr Biol.

[CR10] Godfray HCJ, Beddington JR, Crute IR, Haddad L, Lawrence D, Muir JF, Pretty J, Robinson S, Thomas SM, Toulmin C (2010). Food security: the challenge of feeding 9 billion people. Science.

[CR11] Goettel M, Eing C, Gusbeth C, Straessner R, Frey W (2013). Pulsed electric field assisted extraction of intracellular valuables from microalgae. Algal Res.

[CR12] Harnedy PA, FitzGerald RJ (2011). Bioactive proteins, peptides, and amino acids from macroalgae. J Phycol.

[CR13] Harnedy PA, FitzGerald RJ (2013). Extraction of protein from the macroalga Palmaria palmata. LWT—Food Sci Technol.

[CR14] Holdt S, Kraan S (2011). Bioactive compounds in seaweed: functional food applications and legislation. J Appl Phycol.

[CR15] Joubert Y, Fleurence J (2008) Simultaneous extraction of proteins and DNA by an enzymatic treatment of the cell wall of *Palmaria palmata* (Rhodophyta). J Appl Phycol 20:55–61

[CR16] Jung KA, Lim S-R, Kim Y, Park JM (2013). Potentials of macroalgae as feedstocks for biorefinery. Bioresour Technol.

[CR17] Polikovsky M, Fernand F, Sack M, Frey W, Mueller G, Golberg A (2016) Towards marine biorefineries: energy efficient proteins extractions from marine macroalgae *Ulva lactuca* with pulsed electric fields. Innov Food Sci Emerg Technol. 10.1016/j.ifset.2016.03.013

[CR18] Postma PR, Miron TL, Olivieri G, Barbosa MJ, Wijffels RH, Eppink MH (2015). Mild disintegration of the green microalgae *Chlorella vulgaris* using bead milling. Bioresour Technol.

[CR19] Postma PR, Pataro G, Capitoli M, Barbosa MJ, Wijffels RH, Eppink MH, Olivieri G, Ferrari G (2016). Selective extraction of intracellular components from the microalga *Chlorella vulgaris* by combined pulsed electric field–temperature treatment. Bioresour Technol.

[CR20] Reddy CRK, Fujita Y (1991). Regeneration of plantlets from *Enteromorpha* (Ulvales, Chlorophyta) protoplasts in axenic culture. J Appl Phycol.

[CR21] Reddy CRK, Dipakkore S, Kumar GR, Jha B, Cheney DP, Fujita Y (2006). An improved enzyme preparation for rapid mass production of protoplasts as seed stock for aquaculture of macrophytic marine green algae. Aquaculture.

[CR22] Stegenga H, Mol I (1983). Flora van de Nederlandse zeewieren.

[CR23] Stegenga H, Karremans M, Simons J (2007). Zeewieren van de voormalige oesterputten bij Yerseke. Gerteria.

[CR24] Sun L, Wang S, Gong X, Zhao M, Fu X, Wang L (2009). Isolation, purification and characteristics of R-phycoerythrin from a marine macroalga *Heterosiphonia japonica*. Protein Expr Purif.

[CR25] Tan IH, Blomster J, Hansen G, Leskinen E, Maggs CA, Mann DG, Sluiman HJ, Stanhope MJ (1999). Molecular phylogenetic evidence for a reversible morphogenetic switch controlling the gross morphology of two common genera of green seaweeds, *Ulva* and *Enteromorpha*. Mol Biol Evol.

[CR26] United Nations, Department of Economic and Social Affairs, Population Division (2015) World population prospects: the 2015 revision, key findings and advance tables. Working paper no. ESA/P/WP241

[CR27] van den Burg S, Stuiver, M, Veenstra F, Bikker P, López Contreras A, Palstra A, Broeze J, Jansen H, Jak R, Gerritsen A, Harmsen P, Kals J, Blanco A, Brandenburg W, van Krimpen M, vanDuijn A-P, Mulder W, van Raamsdonk L (2012) A triple preview of the feasibility of sustainable offshore seaweed production in the North Sea. Wageningen UR (University & Research Centre), Wageningen. LEI Report 13-077, pp 1-106

[CR28] van der Wal H, Sperber BLHM, Houweling-Tan B, Bakker RRC, Brandenburg W, López-Contreras AM (2013). Production of acetone, butanol, and ethanol from biomass of the green seaweed *Ulva lactuca*. Bioresour Technol.

[CR29] van Hal JW, Huijgen WJJ, López-Contreras AM (2014). Opportunities and challenges for seaweed in the biobased economy. Trends Biotechnol.

[CR30] Vanthoor-Koopmans M, Wijffels RH, Barbosa MJ, Eppink MHM (2013). Biorefinery of microalgae for food and fuel. Bioresour Technol.

[CR31] Wells ML, Potin P, Craigie JS, Raven JA, Merchant SS, Helliwell KE, Smith AG, Camire ME, Brawley SH (2017). Algae as nutritional and functional food sources: revisiting our understanding. J Appl Phycol.

[CR32] Wijffels RH, Barbosa MJ, Eppink MHM (2010). Microalgae for the production of bulk chemicals and biofuels. Biofuels Bioprod Biorefin.

