# Supplementary material for: Biorefinery of the macroalgae Ulva lactuca: extraction of proteins and carbohydrates by mild disintegration
Source: J Appl Phycol. 2017 Oct 28;30(2):1281–93. doi: 10.1007/s10811-017-1319-8 (PMC5928186; doi:10.1007/s10811-017-1319-8)
Supplement: Supplementary file 1 — (DOCX 17 kb) [file 10811_2017_1319_MOESM1_ESM.docx]

# Supplementary material: Model fit and analysis

A second order polynomial model was created for the protein yield (Eq. (5)) and a third order model for the carbohydrate yield (Eq. (6)):

| $Yp=29.1061-3.42122\cdot X_{1}+2.26359\cdot X_{2}-9.50065\cdot X_{3}-$ $3.46274\cdot{X_{1}}^{2}-0.723018\cdot X_{1}\cdot X_{2}+4.38699\cdot X_{2}\cdot X_{3}$ | (5) |
| --- | --- |
| $Yc=33.9786-2.86699\cdot X_{1}+5.07042\cdot X_{2}-13.0999\cdot X_{3}-1.70571\cdot X_{1}^{2}+1.57841\cdot{X_{2}}^{2}-3.57343\cdot X_{1}\cdot X_{2}+2.38353\cdot X_{2}\cdot X_{3}+2.94412\cdot{X_{2}}^{3}$ | (6) |

Two tests were performed to confirm model validity. The first test is an ANOVA test and is significant at a p value < 0.05. The second test is a lack of fit (LOF) test which passed at a p value > 0.05. An overview of the ANOVA and LOF is provided in Table S.1. For the protein model, both tests were passed and resulted in a model with an r^2^ of 0.609. However, for the carbohydrate model, the model is significant (r^2^ of 0.629), but there is still an LOF. This LOF could be artificial since the reproducibility is close to unity resulting in a very small replicate error. The LOF can especially be noticed at low rotor speeds in which the system was not able to disintegrate the macroalgae thallus (Figures S.1B and S.1C). Therefore, this model should be interpreted as indicative rather than actually predictive.

Table S.1 ANOVA table of MODDE model

| Protein yield | DF | SS | MS (variance) | F | p | SD |
| --- | --- | --- | --- | --- | --- | --- |
| Total | 32 | 28347,4 | 885,857 |  |  |  |
| Constant | 1 | 21220,6 | 21220,6 |  |  |  |
|  |  |  |  |  |  |  |
| Total Corrected | 31 | 7126,84 | 229,898 |  |  | 15,1624 |
| Regression | 6 | 4343,62 | 723,936 | 6,50267 | 0,000 | 26,9061 |
| Residual | 25 | 2783,22 | 111,329 |  |  | 10,5513 |
|  |  |  |  |  |  |  |
| Lack of Fit | 21 | 2658,57 | 126,599 | 4,06237 | 0,091 | 11,2516 |
| (Model Error) |  |  |  |  |  |  |
| Pure Error | 4 | 124,655 | 31,1637 |  |  | 5,58245 |
| (Replicate Error) |  |  |  |  |  |  |
|  |  |  |  |  |  |  |
|  | N = 32 | Q2 = | 0,529 | Cond. no. = | 2,772 |  |
|  | DF = 25 | R2 = | 0,609 | RSD = | 10,55 |  |
|  | Comp. = 2 | R2 Adj. = | 0,516 |  |  |  |
|  |  |  |  |  |  |  |
| Carbohydrate yield~ | DF | SS | MS (variance) | F | p | SD |
| Total | 32 | 51835 | 1619,84 |  |  |  |
| Constant | 1 | 36677,8 | 36677,8 |  |  |  |
|  |  |  |  |  |  |  |
| Total Corrected | 31 | 15157,1 | 488,94 |  |  | 22,112 |
| Regression | 8 | 9536,93 | 1192,12 | 4,87859 | 0,001 | 34,527 |
| Residual | 23 | 5620,2 | 244,356 |  |  | 15,6319 |
|  |  |  |  |  |  |  |
| Lack of Fit | 19 | 5602,49 | 294,868 | 66,5944 | 0,000 | 17,1717 |
| (Model Error) |  |  |  |  |  |  |
| Pure Error | 4 | 17,7113 | 4,42782 |  |  | 2,10424 |
| (Replicate Error) |  |  |  |  |  |  |
|  |  |  |  |  |  |  |
|  | N = 32 | Q2 = | 0,517 | Cond. no. = | Infinite |  |
|  | DF = 23 | R2 = | 0,629 | RSD = | 15,63 |  |
|  | Comp. = 2 | R2 Adj. = | 0,500 |  |  |  |
|  |  |  |  |  |  |  |
